# The WNT Framework in Shaping Immune Cell Responses During Bacterial Infections

**DOI:** 10.3389/fimmu.2019.01985

**Published:** 2019-08-21

**Authors:** Tanushree Mukherjee, Kithiganahalli Narayanaswamy Balaji

**Affiliations:** Department of Microbiology and Cell Biology, Indian Institute of Science, Bangalore, India

**Keywords:** Wnt, infectious diseases, epigenetics, nuclear Wnt signaling, therapeutics

## Abstract

A large proportion of the world is inflicted with health concerns arising from infectious diseases. Moreover, there is a widespread emergence of antibiotic resistance among major infectious agents, partially stemming from their continuous dialog with the host, and their enormous capacity to remodel the latter toward a secure niche. Among the several infection-driven events, moderation of WNT signaling pathway has been identified to be strategically tuned during infections to govern host-pathogen interactions. Primarily known for its role in arbitrating early embryonic developmental events; aberrant activation of the WNT pathway has also been associated with immunological consequences during diverse patho-physiological conditions. Here, we review the different mechanisms by which components of WNT signaling pathways are exploited by discrete bacterial agents for their pathogenesis. Furthermore, recent advances on the cross-talk of WNT with other signaling pathways, the varied modes of WNT-mediated alteration of gene expression, and WNT-dependent post-transcriptional and post-translational regulation of the immune landscape during distinct bacterial infections would be highlighted.

## Introduction

### The Discovery of WNT Ligands and Receptors

Three decades have passed since Dr. Roel Nusse cloned and characterized the first mammalian Wnt gene in 1982. This continued with the congregation of additional 18 ligands and 10 receptors into the large family of Wnt genes. The series of these discoveries have been elaborated by Dr. Nusse in his essay commemorating 30 years of Wnt genes (1). Wnt derives its name from the amalgamation of two lines of research being pursued in the 1980s. On one hand, in an attempt to identify the genes that induced mammary tumors upon infection with mouse mammary tumor virus (MMTV), Nusse and Varmus discovered a single “integration site” that could lead to disease phenotype, and named the same as *int1*. Later it was shown that overexpression of *int1* is sufficient to induce tumorigenesis in mice. At a similar time, *int1* homolog *Wingless* was identified in a Drosophila mutant screen; wherein the gene was found to be essential for segment polarity and for the formation of wing tissue. By the end of 1980s, enormous screens were carried out to decipher other integration sites that may contribute to tumors (*int2, int3, int4*). However, those tumorigenic integration events were diverse and had least homology with *int1*; necessitating the nomenclature to be revisited. The researchers working then with *int1* and *Wingless* consented to the hybrid name “Wnt” (Wingless-related integration site), and *int1* as the pioneer was called Wnt1; whereas *int2* is known today as FGF3, *int3* as NOTCH4 and *int4* as Wnt3A (1). The Wnt family has only been expanding with the youngest member being added to the list in 2002 (2). In view of simplification, the Wnt members and their signaling mechanisms have now been broadly categorized into canonical, non-canonical and alternate, as briefly discussed in the following section.

### The Wnt Signaling Pathway

**Figure 1:**
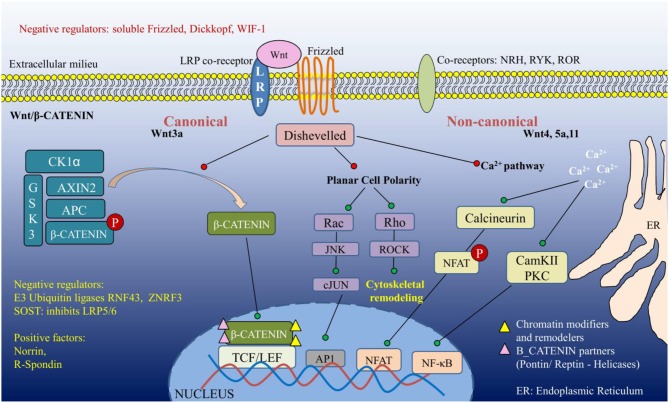
An overview of Wnt signaling pathway.

The Wnt pathway is evolutionarily conserved in all metazoans, where it contributes to critical fate decisions such as polarity, axis formation, organogenesis, tissue homeostasis, and stem cell renewal. Wnt ligands (19 in number), secretory glycoproteins, upon being translated undergo palmitoylation by the ER-resident *Porcupine* acyl transferases, and are then transported through the Golgi network to be docked onto the plasma membrane. When stimulated, the extracellular domain of Wnt is cleaved to release the bioactive ligand into the extracellular milieu, which binds to corresponding Frizzled (Fzd) receptors (10 in humans) in an autocrine or paracrine manner. Fzd form a class of seven-pass transmembrane proteins, showing topological homology to G-protein coupled receptors (GPCRs). The ligand binding occurs at the N-terminal cysteine-rich extracellular domain, bringing about conformational alterations that subsequently activate the adaptor molecule *Disheveled* (Dsh/Dvl). The phosphoprotein Dvl is activated by kinases such as Casein Kinase1, Casein Kinase2, and Protein Kinase C (PKC) among others. Apart from Wnt and Fzd, co-receptors (lipoprotein receptor-related protein, LRP5/6) are critical for tuning the transduction of signal through Wnt receptor. Physiological and biochemical characterization of Wnt ligands, their receptors, co-receptors and the corresponding signaling mechanisms have identified at least three distinguishable consequences of Wnt interaction with specific Frizzled (Fzd) receptors: the canonical β-CATENIN-dependent signaling pathway; and the non-canonical β-CATENIN-independent Planar Cell Polarity and Wnt/Ca^2+^ pathways. Further, the signaling cascades are kept under tight control by various negative regulators operating both intracellularly and extracellularly (briefly described in Figure 1). Owing to the modular architecture, Dvl stands as a single molecule past the ligand-receptor interface to be common in all the three pathways (3–5).

#### Wnt/β-CATENIN Signaling Pathway

This forms the first pathway to be identified by genetic screens in Drosophila and validated in worms, frogs, fishes, and mice. The central objective of the pathway ensures the stabilization and activation of the transcription co-activator β-CATENIN. When unstimulated, β-CATENIN is trapped in a destruction complex composed of Axin, Adenomatous Polyposis Coli (APC), Casein kinase1α (CK1α), glycogen synthase kinase3 (GSK3). Here, GSK3/CK1α phosphorylates β-CATENIN, thereby priming it for ubiquitination-mediated proteasomal degradation. Upon signaling activation, Axin is translocated to the membrane to bind to LRP5/6, which leads to a series of relatively uncharacterized events activating Dvl and inactivating GSK3. These steps release the repression from β-CATENIN and allow it to translocate to the nucleus along with specific nuclear localization signal (NLS)-containing proteins. β-CATENIN, then effectuates Wnt-specific gene expression by interacting with the TCF/LEF (T cell factor/ lymphoid enhancer factor) family of DNA-binding transcription factors, while eliminating the negative regulator Groucho from the site.

#### Planar Cell Polarity (PCP) Pathway

The non-canonical pathways activate Fzd and utilize specific domains of Dvl to transduce the signals. LRP5 and LRP6 do not participate in the non-canonical pathways, and are reportedly replaced by NRH1, Ryk, Ror as co-receptors. In the PCP pathway, activated Dvl adopts a route through GTPases Rho and Rac to activate ROCK and JNK, respectively. These events majorly culminate in cytoskeletal re-organization, and mechanisms of the pathway's contribution to transcriptional rewiring still require investigation.

#### Wnt/Ca^2+^ Pathway

This pathway came to light with the observation that discrete Wnt signals can lead to Ca^2+^ release from the endoplasmic reticulum (ER) in a GPCR-dependent manner. Though the precise GPCR has not been elucidated, it is clear that the activated trimeric G proteins lead to Ca^2+^ release and activation of Ca^2+^-dependent kinases such as Calcium/ calmodulin Kinase II (CamKII) and PKC, and transcription factor NFAT. Interestingly, CamKII can also intercept β-CATENIN-driven regulation of gene expression, thereby altering the canonical arm of Wnt signaling.

## Wnt Pathway in Infections

Conventionally, Wnt genes have been correlated with carcinogenesis, retrovirus-mediated tumorigenesis and developmental processes, however, its contributions in the context of various infections is manifold (5). Sentinel macrophages, epithelial cells and other immune cells adopt a number of defense mechanisms to defy the invading pathogens that include autophagy, apoptosis, inflammation, antigen presentation, ER-stress regulation, inflammasome activation, and priming of cell-mediated immune responses such as T helper or cytotoxic phenotype. This review will give a brief account of the same with respect to bacterial infections in the light of Wnt signaling pathways.

### Regulation of Inflammation and Adaptive Immune Responses During Bacterial Infections

Inflammation is a complex process encompassing the differential activation of pathways leading to the expression and secretion of a set of cytokines by the host cells themselves or by host cell-mediated priming of the adaptive immune system. Skewing cytokine response to establish successful infection is adopted by several pathogens such as *Mycobacterium tuberculosis, Shigella flexneri, Salmonella typhimurium, Citrobacter rodentium* etc. In this context, it was recently shown that canonical Wnt signaling is essential to maintain an inflammatory equipoise during *S. flexneri* infection. It was found that *Shigella*-induced inflammation is maintained by NOD2-driven Epidermal Growth Factor Receptor (EGFR)-dependent activation of Wnt/β-CATENIN axis, leading to the production of the anti-inflammatory molecule IDO1 (indoleamine 2, 3 dioxygenase1). The perturbation of Wnt signaling compromised IDO1 expression and pharmacological inhibition of IDO1 in mice exacerbated Shigellosis by inducing intestinal hyper-inflammation (6). Another instance of NOD2-driven inflammation was replicated in an acute arthritis model wherein Singh et al. utilized NOD2 agonist muramyl dipeptide (MDP) to demonstrate Wnt/β-CATENIN activation through LYPD6. It was found that the resultant Wnt signaling orchestrates β-CATENIN-dependent XIAP expression that mediated NLRP3 inflammasome activation, Caspase1 maturation and IL-1β secretion, thereby leading to the development of the inflammatory condition of acute arthritis in mice (7). Therefore, in contrasting conditions the same components of the canonical Wnt pathway generate intermediates that influence net inflammation.

Apart from the direct interception of Wnt pathway, certain pathogens alter Wnt signaling events by modulating the expression of the regulators of the pathway. For instance, in a model of *C. rodentium* infection in resistant C57BL/6J mice vs. susceptible C3H/HeOuJ and FVB mice, the authors found a robust expression of R-Spondin (Rspo2) in the susceptible strains (8). Rspo2 is known to activate Wnt pathway by negatively regulating E3 ubiquitin ligases ZNF43 and ZNRF3 that allow proteasomal degradation of Wnt pathway components. The authors show that Rspo2 is associated with pathological Wnt/β-CATENIN activation-driven loss of intestinal differentiation, resulting in susceptibility to *C. rodentium* infection. Further studies by the same group found Rspo2 to be associated with enhanced MHC-II-driven antigen presentation and generation of Th1/ Th17 in susceptible mice (9). This could be attributed to the pronounced ability of proliferating intestinal cells to sense the pathogen because of an apparent loss of functionally differentiated goblet cells in Rspo2-driven Wnt active intolerant/susceptible mice.

### Modulation of Host Cell Death Pathways

The regulated execution of specific cell death pathways enables bacterial pathogens to either localize in host cells or disseminate systemically during the course of infection. Therefore, it can be surmised that the dynamics of these pathways would be carefully tapped during host-pathogen interactions. Wnt signaling has been significantly implicated in apoptotic events during development, and has subsequently been found to contribute to distinct cell death phenomena associated with infections. It has been reported that canonical Wnt3a-β-CATENIN signaling enhances apoptotic host cell death during *Mycobacterium bovis* BCG (BCG) infection via mitochondrial cytochrome c-driven Caspase3 activation. This involved the upregulation of the pro-apoptotic Bax and downregulation of anti-apoptotic Mcl1 proteins (10). Alongside, another study reported the ability of Wnt3a to inhibit BCG-induced necrosis (another cell death pathway) by restricting the ROS-dependent PARP1-AIF cascade (11). These studies employed the vaccine strain BCG, which might present immunologically diverse regulatory outcomes compared to virulent mycobacteria. However, the parallel results of enhanced apoptosis and restricted necrosis may be explained by the ability of BCG to utilize Wnt pathway-driven apoptosis as a strategy to establish infection at early stages and therefore the dissemination-prone necrotic pathway is limited. In another instance, Liu et al. demonstrated that *Salmonella* infection enhances Wnt2 expression at the transcript and protein level, partially by AvrA-mediated regulation, which is responsible for inhibiting *Salmonella* infection-driven apoptotic/necrotic host cell death, thereby enabling pathogen survival (12). Recent advances have identified multiple forms of regulated cell death mechanisms, apart from apoptosis, such as RIPK1-RIPK3-MLKL-dependent necroptosis and iron-mediated ferroptosis, however, these have not been explored in the context of Wnt signaling and infections.

### Wnt-Dependent Regulation of Cellular Homeostatic Processes

Among different cellular events, autophagy forms a homeostatic process initiated to unload host cells of non-functional/unfolded proteins, worn-out/excess organelles and exogenous agents, including intracellular pathogens. The processing of intracellular pathogens, referred to as xenophagy, displays an efficient host response to infections. The implication of Wnt pathway in the inhibition of autophagy in the context of mycobacterial infection has been demonstrated. It was found that infection with pathogenic bacteria, including BCG, *S. flexneri* and *Listeria monocytogenes* stimulate the expression of Wnt ligand Wnt5a, receptor Fzd4 and co-receptor Lrp5 to bring about the inhibition of IFNγ-induced autophagy. The detailed molecular mechanism implied an mTOR-dependent miR31- and miR155-driven downregulation of the phosphatase PP2A, thereby stabilizing the inhibitory phosphorylation on the negative regulator of Wnt pathway i.e., GSK3β. However, non-pathogenic bacteria such as *E. coli* failed to execute the string of events (13). Interestingly, in another study with *Ehrlichia chaffeensis*, Lina et al. demonstrated the ability of the pathogen to utilize Wnt-driven mTOR-PI3K pathway to inhibit autophagy by restricting the fusion of amphisomes (bacteria-containing endosomes decorated with autophagy markers BECLIN1, p62, LC3BII) with lysosomes. The authors suggest that the virulence factor Etf1 allows amphisome formation in order to acquire nutrients, while, another set of virulence determinants TRP120, 32 and 47 act in concert to activate Dvl/Wnt pathway to mediate mTOR-driven TFEB cytosolic retention and consequent inhibition of amphisome-lysosome fusion (14). These studies clearly indicate the ability of pathogens to hijack the Wnt pathway to evade host innate defense of autophagy. In addition to these reports, Jati et al. demonstrated that pathogens such as *Pseudomonas aeruginosa* and *Streptococcus pneumoniae* indeed downregulate Wnt5a in order to suppress autophagy and establish a successful infection (15). Thus, Wnt signaling is regulated disparately by diverse pathogens to execute similar immune responses, indicating toward the specificity of virulence mechanisms employed by infectious agents.

### Non-canonical Wnt in Infectious Diseases

The non-canonical components of the Wnt pathway have distinct roles in patho-physiologies, and their association with infectious diseases has started to garner significant attention. For instance, Luo et al. investigated the ability of the obligate intracellular bacterium *E. chaffeensis*, causing human monocytotrophic ehrlichiosis (HME) to utilize the non-canonical Wnt pathway for its pathogenesis. The authors showed that the unusual bacterium upregulates several components of the Wnt pathway, including ligands Wnt6 and 10a; receptors Fzd5 and 9; co-receptor LRP6 and TCF7 early during infection. Interestingly, *E. chaffeensis* tandem repeat proteins (TRPs) were shown to assist bacterial internalization through non-canonical Wnt components, where they interacted with Wnt pathway regulators like ARID2, KDM6B, IRF2BP2, PPP3R1, and VPS29, thus influencing Wnt signaling activation and aiding in bacterial survival by both canonical β-CATENIN and non-canonical Ca^2+^-CAMKII-NFAT routes (16). Apart from this, several reports have focused on enteric pathogens for their association with the non-canonical Wnt pathway. Wnt11 has been reported to be enhanced during intestinal bowel disease (IBD), and Liu et al. demonstrated its relevance in the context of bacterial inflammation for the first time. They found that infection of host epithelial cells with *S. typhimurium* leads to an elevated expression of Wnt11, dependent on the *Salmonella* effector protein AvrA. Further, the *in vivo* studies showed a relocalization of Wnt11 to the lower crypts during infection and also demonstrated that overexpressing Wnt11 shows inhibitory effects on *Salmonella* internalization and IL-8 expression-mediated intestinal inflammation (17). Another instance is detailed with the kinome array conducted for *Salmonella enteritidis* infection in chicken, wherein the authors find an infection-driven activation of non-canonical components of the Wnt pathway in Fzd1-dependent manner. This was shown to converge to the phosphorylation of Fzd1-CamKII/β-CATENIN/Protein Kinase C that eventually activates NFAT and suppresses canonical Wnt signaling. This aids the expression of anti-inflammatory IL-10 and TGFβ, thereby generating a tolerogenic state and allowing persistent colonization of *Salmonella* in the chicken gut (18). In the realm of mycobacterial infection, the contribution of canonical Wnt/β-CATENIN pathway has been detailed in several studies (13, 19). Interestingly, mycobacterial infection has also been reported to mediate the expression of non-canonical Wnt components (Wnt5a and Wnt6). In one instance, Wnt5a-Fzd5 axis was shown to be activated in myeloid cells of the TB lesion in order to prime cell-mediated pro-inflammatory immune responses such as IFNγ production from T cells (20). Conversely, in another study, Wnt6 was found to be expressed specifically by lipid-laden macrophages in TB granulomas and was associated with Arginase1 expression, while being negatively correlated with pro-inflammatory TNFα production (21). Although both the studies were conducted in the mouse model of chronic TB infection, it appears that the cell type expressing specific Wnt ligands and their spatio-temporal abundance strongly influences the immune cell polarization and the net immune outcome.

### Cross-Talk of Wnt With Other Signaling Pathways During Infections

Infection with pathogens triggers several signaling pathways that act in concert to define the immune outcome. The Wnt cascade itself establishes communication with intracellular signaling intermediates in physiological and infectious scenarios. This is achieved by the presence of common upstream regulators or downstream target molecules. It has been shown that Wnt interacts with Sonic Hedgehog (SHH) signaling through GSK3β, which acts as a negative regulator of both the pathways. In the context of mycobacterial infection in macrophages, miRNAs 31 and 155 were found to repress the expression of the phosphatase PP2A, thereby retaining the inhibitory phosphorylation of GSK3β, and consequently allowing the activation of Wnt and SHH through β-CATENIN and NUMB, respectively (13). Further, in another study, a pathogenic encounter with Mtb, *Salmonella* or *Staphylococcus* was found to induce both NOTCH (Notch1, Jagged1) and Wnt (Wnt5a, Fzd4, Lrp5) signaling pathways. This pathogen-specific TLR2-dependent activation of Wnt and NOTCH relied on a Wnt/ β-CATENIN-driven expression of NOTCH pathway ligand Jagged1 in iNOS/NO-dependent manner. Critically, such studies broaden the repertoire of signals responsible for canonical β-CATENIN activation to include non-canonical Wnt ligands, such as WNT5a. Such an orchestration was observed to mediate regulatory T cell expansion during pathogenic bacterial infections (19). The signaling cross-talks are witnessed across phyla and species, ranging from Drosophila and zebrafish to mice and pigs. In this light, a study by Huan et al. showed the synergistic effects of Wnt attenuation (assessed by cytosolic localization of β-CATENIN and downregulation of target gene expression) and NOTCH pathway activation in regulating goblet cell maturation, mucosal integrity and crypt cell proliferation in pigs infected with *Lawsonia intracellularis*, thus providing insights into the pathogenesis of this bacterial agent (22).

Recent reports demonstrate Wnt pathway to respond to *S. flexneri* infection-activated EGFR pathway. It was shown that the pathogen utilizes EGFR pathway to initiate a cascade comprising the engagement of c-Abl and HIPK2 to mediate the activation of the Wnt/ β-CATENIN axis and the subsequent expression of anti-inflammatory IDO1 (6). In the context of another intestinal infection with *C. rodentium*, Wnt signaling has been reported to coordinate with AHR signals to maintain a balance of intestinal stem cell renewal and differentiation. Metidji et al. demonstrated that AHR pathway transcriptionally upregulates Wnt negative factors RNF43 and ZNRF3, thereby limiting Wnt signaling activation. This check on Wnt pathway allows intestinal stem cell repair and differentiation; and prevents from intestinal infections (*C. rodentium*) and formation of colorectal cancers (23).

### Hijacking Wnt Components for Gaining Access to Host Cells

The intracellular events post-receptor-driven activation of Wnt pathway are well appreciated. The independent roles for the receptors themselves during infections form a relatively recent development in the functional spectra of this signaling cascade. In 2016, Tao et al. demonstrated the implication of Fzd receptors in the invasion and thereby pathogenesis of the Gram positive bacterium *Clostridium difficile*. They found that the pertinent TcdB toxin of the pathogen is infused into host cells via Fzd2 receptor, alongside Fzd1 and Fzd7. Single knockout of either Fzd1/2/7 or triple knockout in HeLa cells compromised TcdB-mediated host cell rounding and cell death; while complementing any of the three Fzd in the triple knockout conditions restored the host cell sensitivity to the toxin. Having validated the same in colorectal cancer cell line HT-29 and colonic organoids and having confirmed its relevance in detecting TcdB in mouse colon epithelium *in vivo*, it is intriguing to consider Fzd receptors as potential targets for minimizing the detrimental effects of this infection (24). Another interesting study by Zhang et al. (25), demonstrated the effect of Axin1 in *Salmonella* invasion and resultant inflammation. They found that the pathogen attaches to the host cell membrane irrespective of the presence of Axin1; however, the invasion of *Salmonella* is severely compromised in cells overexpressing Axin1. Mechanistically, the authors found that *Salmonella* strategizes the depletion of Axin1 early during infection by post-translational mechanisms through ubiquitination and SUMOylation (25). Of note is that a proportion of overexpressed Axin1 is found localized to the plasma membrane upon *Salmonella* infection, suggesting a possible inhibitory effect being generated at the entry point.

### Wnt-Dependent Epigenetic Regulation of Infection-Driven Immune Responses

The paradigm of regulation of gene expression also includes accurate and swift alterations mediated by epigenetic mechanisms. These epigenetic changes result from the covalent modifications of DNA/ histones or the structural reorganization of chromatin. The bearings of such modifications are immense, as recruitment of the transcriptional co-activator, β-CATENIN, alone is not sufficient to orchestrate WNT target gene expression. Therefore, a major focus of the current research invests in exploring the epigenetic interventions. In this front, it is known that upon nuclear translocation, β-CATENIN interacts with multiple factors through its N- and C-terminal domains. The N-terminus interacts with LEF to allow its DNA binding, while also associating with a number of factors such as Legless (Lgl) and Pygopus (Pygo). The C-terminus is more diverse in the repertoire of chromatin binding proteins that include histone methyl transferases such as MLL, PRC2 complex (EZH2), demethylase LSD1, histone acetyl transferase like p300, and chromatin remodelers such as BRG1 and ISW1. A study by Yakulov et al. has compared the chromatin partners of β-CATENIN in the presence or absence of the canonical ligand, WNT3a (26, 27). Although, some of these association studies have been prominent in cancers and development, the implications of the same in bacterial infections is in its infancy.

In a model of *C. rodentium* infection in mice, Roy et al. proposed the activation of WNT signaling-driven crypt hyperplasia and tumorigenesis to result from EZH2-dependent downregulation of the negative Wnt regulator WIF1. They demonstrate an enhanced expression of EZH2 upon infection with *Citrobacter* (28). However, there is a lack of evidences that compare the infection-moderated chromatin profile or alterations of associating partners of β-CATENIN. Such instances may be borrowed from studies elucidating distinct pathologies. For instance, the type-II protein arginine methyltransferase, PRMT5, activates Wnt signaling and Wnt/ β-CATENIN target gene expression by differentially modulating the promoter occupancy by co-activators and co-repressors in lymphoma cells. Chung et al. specifically demonstrated that PRMT5 inhibition reduces the recruitment of co-activators p300, MLL1, while enhancing the co-repressors HDAC2 and LSD1 in lymphoma cell lines. Interestingly, the occupancy of β-CATENIN over the promoters of these target genes does not alter significantly upon PRMT5 inhibition making it an interesting supposition to assess if PRMT5 associates with β-CATENIN to mediate differential recruitment of activators/repressors in Wnt-specific gene expression even during infections (29).

### Future Perspectives

Infectious diseases account for a vast proportion of disability associated life years (DALYs) as well as mortality in the global context. This stems from the ability of major infectious agents to adopt strategies in order to co-evolve with their host, and exhibit immune evasion and subversion mechanisms as a part of their survival tactics. With the early reports of the implications of Wnt pathway in development and tumorigenesis, it could be foreseen that the pathway might be hijacked by infectious entities to disrupt homeostasis and establish infection. In this light, we discussed the many facets that are exploited by pathogens along the Wnt signaling axis, including Wnt ligands, receptors, coreceptors, transcription factors, and certain epigenetic alterations (summarized in Table 1). However, the increasing complexities being identified for the Wnt pathway provides an array of avenues that still remain untapped in the realm of bacterial infections.

**Table 1:** Interactions of bacterial pathogens with Wnt pathway components.

**Pathogens**	**WNT component involved**	**Effect on infection-driven processes**	**References**
*Shigella flexneri*	Canonical β-CATENIN pathway	Maintaining inflammatory equipoise through the regulation of IDO1	(6)
*Citrobacter rodentium*	R-spondin 2-mediated Wnt activation	Involved in loss of intestinal differentiation	(8)
		Leads to increased MHC-II responses and Th1/17-driven hyper-inflammation	(9)
*Mycobacterium bovis* BCG	Wnt3a	Induction of apoptosis	(10)
*Mycobacterium bovis* BCG	Wnt3a	Inhibition of necrosis	(11)
*Salmonella*	Wnt2	Inhibition of necrosis	(12)
BCG, *Shigella flexneri, Listeria monocytogenes*	Wnt5a, Fzd4, Lrp5	Inhibition of autophagy	(13)
*Ehrlichia chaffeensis*	Canonical Wnt/Dvl-mTOR axis	Inhibition of autophagy	(14)
*Pseudomonas aeruginosa*/ *Streptococcus pneumoniae*	Downregulation of Wnt5a	Inhibition of autophagy	(15)
*Ehrlichia chaffeensis*	Wnt6, Wnt10a, Fzd5, Fzd9, Lrp6, Tcf7	Assist bacterial internalization and survival	(16)
*Salmonella typhimurium*	Wnt11	Inhibition of *Salmonella* internalization and IL-8 expression-mediated inflammation	(17)
*Salmonella enteritidis*	Fzd1	Production of anti-inflammatory cytokines IL-10, TGFβ	(18)
*Mycobacterium tuberculosis*	Wnt5a-Fzd5	Induction of pro-inflammatory immune response	(20)
*Mycobacterium tuberculosis*	Wnt6	Expressed in lipid droplets and associated with production of anti-inflammatory Arginase1	(21)
*Mycobacterium tuberculosis, Salmonella, Staphylococcus*	Wnt5a, Fzd4, Lrp5	Regulatory T cell expansion	(19)
*Lawsonia intracellularis*	Downregulated Wnt signaling (cytosolic β-CATENIN)	Inhibition of goblet cell maturation at peak of infection	(22)
*Citrobacter rodentium*	Upregulation of negative regulators of Wnt pathway - RNF43 ZNRF3	Intestinal stem cell renewal and differentiation, thereby preventing intestinal infections	(23)
*Clostridium difficile*	Fzd1, 2, 7	Assist in pathogen internalization	(24)
*Salmonella*	Axin1	Limits invasion into host cells	(25)
*Citrobacter rodentium*	Epigenetic downregulation of WIF1, leading to Wnt activation	Crypt hyperplasia and tumorigenesis	(28)

The canonical and non-canonical Wnt signaling pathways are majorly distinguished by the nuclear translocation of the transcriptional co-activator β-CATENIN, which alters immune gene expression. Notably, several other Wnt components such as APC, Axin2, and Dvl proteins harbor nuclear import and export signals and have been found to shuttle to the nucleus in order to fine tune the outcome of the Wnt pathway (30–33). However, it is unclear as to how these cytosolic/membrane-localized Wnt components are targeted to the nucleus. Since the infection outcomes depend on the transcriptional landscape, filling the gaps in nuclear Wnt signaling may assist the understanding of gene expression in a more cohesive fashion. It would be interesting to assess if bacterial pathogens or their virulence factors associate with these accessory components to invade into the host cell nucleus or differentially drive their shuttling to enhance their pathogenesis.

Chromatin modifications contribute significantly in defining the infection-induced transcriptome. With the premise that β-CATENIN is decorated with several chromatin modifiers and remodelers once inside the nucleus, exploring the epigenetics along Wnt signaling axis during bacterial infections, as a cause or consequence, requires further investigation. Moreover, it is reported that certain epigenetic factors can also act on host cytosolic proteins to alter their functions. In a very recent series of research outcomes, it was identified that the arginine methyl transferase PRMT1 is important for Wnt signaling activation. Mechanistically, the asymmetric arginine dimethylation conferred by PRMT1 on many of the GSK3 target proteins either primed them for GSK3-mediated phosphorylation or packaged them along with GSK3 into multi-vesicular bodies (MVBs), which stands as a pre-requisite for β-CATENIN nuclear translocation and Wnt activation (34). Further development showed that PRMT1-Wnt axis is crucial for governing macropinocytosis- (referring to the uptake of extracellular fluid with particles >0.2 μm)-driven endosomal trafficking and lysosomal degradation to increase the bioavailability of free amino acids (35). This attribute of Wnt signaling can be extended to infectious scenarios, where pathogens derive a large portion of nutrients from the host. It might possibly posit a critical persistence mechanism as many of the infections deal with innate sentinels such as macrophages, which sample their microenvironment and may collect nutrients by macropinocytosis upon infection-driven Wnt activation.

Finally, the extensive repertoire of Fzd receptors still require detailed investigation for their possible implications in pathogen uptake and ensuing microautophagy/macropinocytosis events. The endless possibilities of Wnt functions, given the apparent cross-talks and the overlaps of the canonical and non-canonical components, can levy contrasting immune outcomes during host-pathogen interactions as may be observed with the ability of Mtb to induce canonical Wnt/ β-CATENIN signaling to inhibit autophagy, while the non-canonical Wnt being downregulated by *P. aeruginosa* and *S. pneumoniae* infections to execute the same (13, 15). Wnt was primarily recognized to be essential for cell proliferation and differentiation processes, which also included the modulation of host cell death. It may be noted that Wnt-directed host cell death during infections has only been restricted to apoptosis and necrosis. Advances in the understanding have revealed the existence of multiple regulated cell death pathways, such as necroptosis, ferroptosis, and pyroptosis. Though Wnt has been associated with components of these cell-death events, it has not been studied in infections. For instance, necroptosis that occurs via the formation of “necrosome” consisting of RIPK1-RIPK3-MLKL axis has been linked with Wnt pathway. In a study with colorectal cancer, it was shown that RIPK3 knockout leads to the excessive activation of various pathways including Wnt/ β-CATENIN (36). Moreover, recent evidence succinctly demonstrated that GPX4 interacts with canonical Wnt transcription factors TCF3 and TCF4, occupies the promoters of Wnt target genes and suppresses their expression (37). GPX4 acts as a major regulator of ferroptosis (iron-induced programmed cell death) by buffering peroxide levels, and is downregulated by infectious agents like Mtb (38). These observations offer a vast avenue for probing into the mechanistic insights of Wnt-associated cell death events during infections.

**Figure 2:**
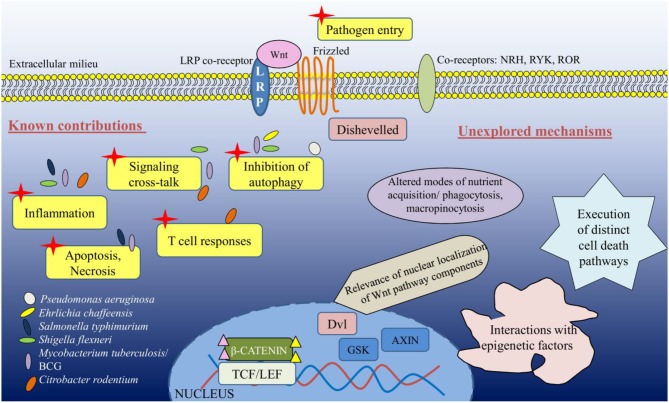
The unexplored facets of Wnt signaling during bacterial infections.

A major proportion of research on Wnt pathway in the realm of infectious diseases limits to intestinal bacterial infections and subsequent inflammation. However, as may be noted now that the potential implications of a versatile and complex pathway like Wnt has so far not been appropriately exploited in distinct infections and associated cellular consequences. With the expanding dynamics of antibiotic resistance among globally relevant pathogens, host-directed therapeutics form an essential alternative (39), and we believe that unraveling the contributions of Wnt pathway would provide critical cues for the same. Several platforms are being synthesized for targeting the Wnt pathway in distinct pathologies, however, it is imperative to understand that the numerous ligand-receptor permutations along with their downstream cross-talks present an unavoidable drawback for Wnt-directed therapeutics (40). Moreover, the ubiquity of kinases like GSK3 would compromise the specificity of such attempts. Overall, though it would be highly challenging to design Wnt-specific therapies, it must be envisioned that a thorough analysis of the pathway with respect to its interactome, including chromatin modifiers/remodelers, cell death factors to name a few, and how they are redefined during infections would offer a novel paradigm for combinatorial adjuncts (Figure 2).

## Author Contributions

KB constructed the framework of the manuscript. TM wrote the initial draft. TM and KB finalized, edited, and proofread the manuscript.

### Conflict of Interest Statement

The authors declare that the research was conducted in the absence of any commercial or financial relationships that could be construed as a potential conflict of interest.
